# Genome-wide transcriptome analysis of the transition from primary to secondary stem development in *Populus trichocarpa*

**DOI:** 10.1186/1471-2164-11-150

**Published:** 2010-03-04

**Authors:** Palitha Dharmawardhana, Amy M Brunner, Steven H Strauss

**Affiliations:** 1 Department of Forest Ecosystems and Society, Oregon State University, Corvallis, OR, 97331-5752, USA; 2 Department of Forest Resources and Environmental Conservation, Virginia Tech, Blacksburg, VA, 24061-0324, USA

## Abstract

**Background:**

With its genome sequence and other experimental attributes, *Populus trichocarpa *has become the model species for genomic studies of wood development. Wood is derived from secondary growth of tree stems, and begins with the development of a ring of vascular cambium in the young developing stem. The terminal region of the developing shoot provides a steep developmental gradient from primary to secondary growth that facilitates identification of genes that play specialized functions during each of these phases of growth.

**Results:**

Using a genomic microarray representing the majority of the transcriptome, we profiled gene expression in stem segments that spanned primary to secondary growth. We found 3,016 genes that were differentially expressed during stem development (Q-value ≤ 0.05; >2-fold expression variation), and 15% of these genes encode proteins with no significant identities to known genes. We identified all gene family members putatively involved in secondary growth for carbohydrate active enzymes, tubulins, actins, actin depolymerizing factors, fasciclin-like AGPs, and vascular development-associated transcription factors. Almost 70% of expressed transcription factors were upregulated during the transition to secondary growth. The primary shoot elongation region of the stem contained specific carbohydrate active enzyme and expansin family members that are likely to function in primary cell wall synthesis and modification. Genes involved in plant defense and protective functions were also dominant in the primary growth region.

**Conclusion:**

Our results describe the global patterns of gene expression that occur during the transition from primary to secondary stem growth. We were able to identify three major patterns of gene expression and over-represented gene ontology categories during stem development. The new regulatory factors and cell wall biogenesis genes that we identified provide candidate genes for further functional characterization, as well as new tools for molecular breeding and biotechnology aimed at improvement of tree growth rate, crown form, and wood quality.

## Background

With the completion of its genome sequence *Populus trichocarpa*, the genus *Populus *(poplar) has become the consensus model taxon for woody plant genomics and biotechnology. Many attributes of *Populus *makes it well suited for genomic research, including facile transformation of selected genotypes; abundant natural genetic diversity; ease of vegetative propagation; rapid growth; interspecific hybrids and mapping pedigrees; availability of genome scale microarrays; and abundant EST resources (reviewed in [1-3]). Although herbaceous short generation models such as Arabidopsis have powerful advantages for studying plant development, including fundamental aspects of lignocellulosic tissue development [4-6], poplar enables a detailed, integrated analysis of secondary growth and other developmental processes such as seasonal dormancy induction whose expression is not fully developed in annual herbaceous species.

The perennial stem growth habit of most tree species is characterized by secondary growth that results in a cumulative increase in girth during each growth cycle. This is achieved by cell division activity of the vascular cambium, with subsequent differentiation of secondary xylem towards the inside of the cambium and secondary phloem towards the outside. This coordinated and environmentally responsive program of meristematic differentiation and tissue patterning involves multiple developmental processes with interacting regulatory mechanisms (reviewed in [7,8,4]).

With the increasing availability of genomic tools, many studies have been conducted to help understand these developmental processes in more depth. Wood development has been characterized by expression profiling in aspen [9-11], *Pinus *[12,13], black locust [14], *Eucalyptus *[15,16] and spruce [17]. These studies, and investigations on vascular cell development in herbaceous species Arabidopsis and *Zinnia*, have identified several classes of important transcription regulators and structural genes involved in secondary cell wall biogenesis (reviewed in [8,18,7,19]). The developmental transition from primary to secondary growth that occurs along the stem has been used as a unique system to identify processes specific to secondary growth in sitka spruce [18] and aspen [20,21]. The aspen studies, based on cDNA-AFLP, characterized 85 and 271 transcript derived fragments, respectively, as differentially expressed during secondary growth, and includes many regulatory and structural genes involved in secondary growth and secondary wall biogenesis. Our study extends this line of work to comprehensively cover the transcriptome of *Populus *during primary to secondary growth transition. We detected more than 36,000 *Populus trichocarpa *genes, and found that more than 3,000 were differentially expressed during stem development. We also provide an in depth look at the expression of several classes of transcription regulators and structural gene families whose functions are closely linked to stem and wood development.

## Results and discussion

### Poplar stem development

To determine transcriptional changes that occur during primary and secondary growth phases of stem development, we analyzed stem segments from successive internodes below the apex (plastochron indices 2, 3, 4, and 5: [22]), and from internode (IN) 9 from further down the stem. IN1 was not analyzed as it was too short to sample without including tissue from the nodes and axillary meristems.

**Figure 1:**
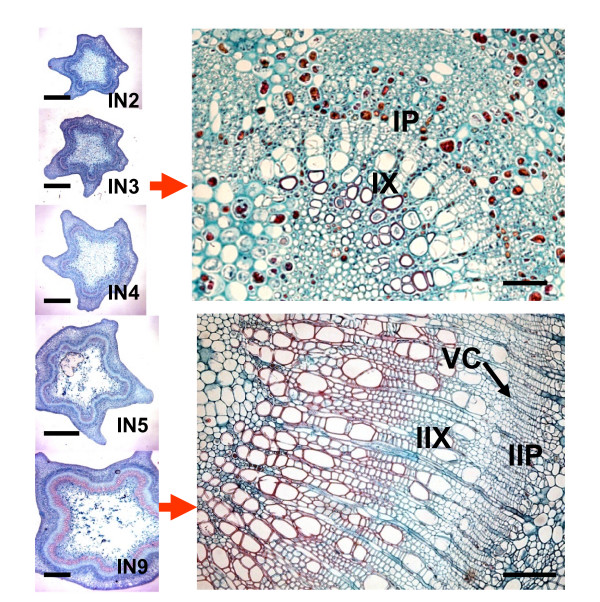
**Anatomy of stem internodes from developing *Populus trichocarpa *Nisqually-1 shoot apex**. IN2, IN3, IN4, IN5 and IN9 are Toluidine Blue-O stained cross sections from stem internodes 2 to 9 (plastochron index) of an actively growing young tree under field conditions in Corvallis, Oregon. The IN3 stem section shows well developed primary vascular tissue. IN9 shows well developed secondary growth with well lignified xylem tissue. IIP, IIX - secondary phloem and xylem respectively; VC - vascular cambium; IP, IX - primary phloem and primary xylem respectively. Scale bar for IN2-IN9 whole stem cross sections is 0.5 mm; that for IN3 and IN9 stained partial cross sections is 50 um.

**Figure 2:**
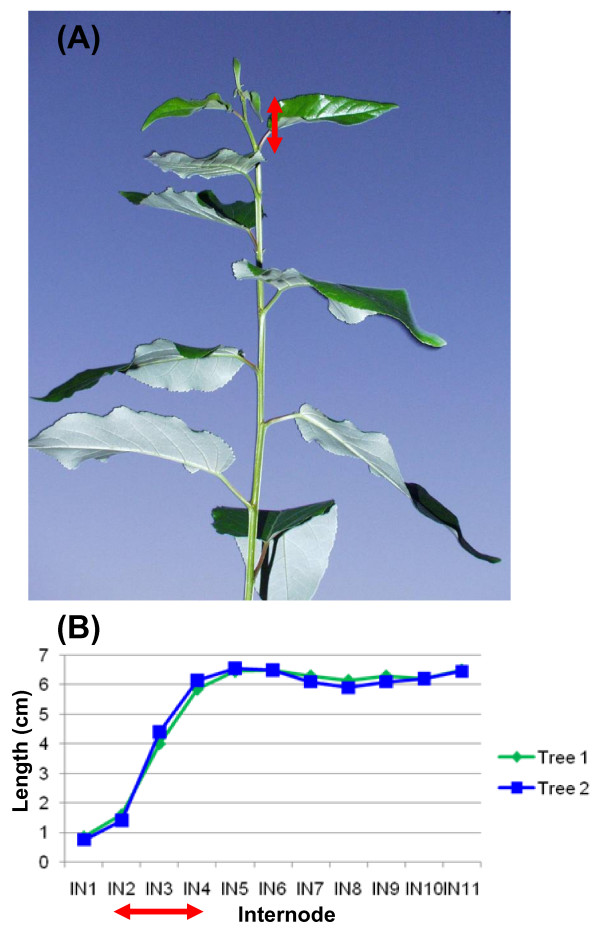
**Apical shoot internode growth of *Populus trichocarpa *Nisqually 1**. (A) elongation zone (red arrow) and (B) change in internode lengths along the developing stem of the two trees sampled for RNA.

The shoot apical meristem of poplar is an indeterminate primary meristem that gives rise to ground tissue and procambium. The vascular tissue of primary growth, primary xylem and primary phloem, develops directly from procambium and is the dominant vasculature in IN2 and IN3 (Figure 1). IN3 shows the first signs of interfascicular parenchyma cells that differentiate into a full ring of cambium initials, enabling radial secondary stem growth. IN5 and IN 9 have well developed secondary phloem tissue and secondary xylem vessels, as well as fibers with well lignified secondary walls (red staining, Figure 1). Figure 2 illustrates that the zone of stem elongation is limited to the terminal internodes up to IN4, which essentially corresponds to the primary stem growth region. With the development of vascular cambium and secondary growth, stem elongation ceases while the girth of the stem axis continues to increase. The other secondary meristem, bark/cork cambium, appears much later during stem development and is not detectably represented in the IN9 sample. Secondary growth in this tissue is associated with production of secondary xylem elements and secondary phloem fibers that have well developed secondary walls. However, although a minor component the primary xylem elements in the primary growth region also have some secondary wall formation and associated lignification (Figure 1, IN3).

### Secondary Xylem Marker Genes are upregulated in IN9

**Figure 3:**
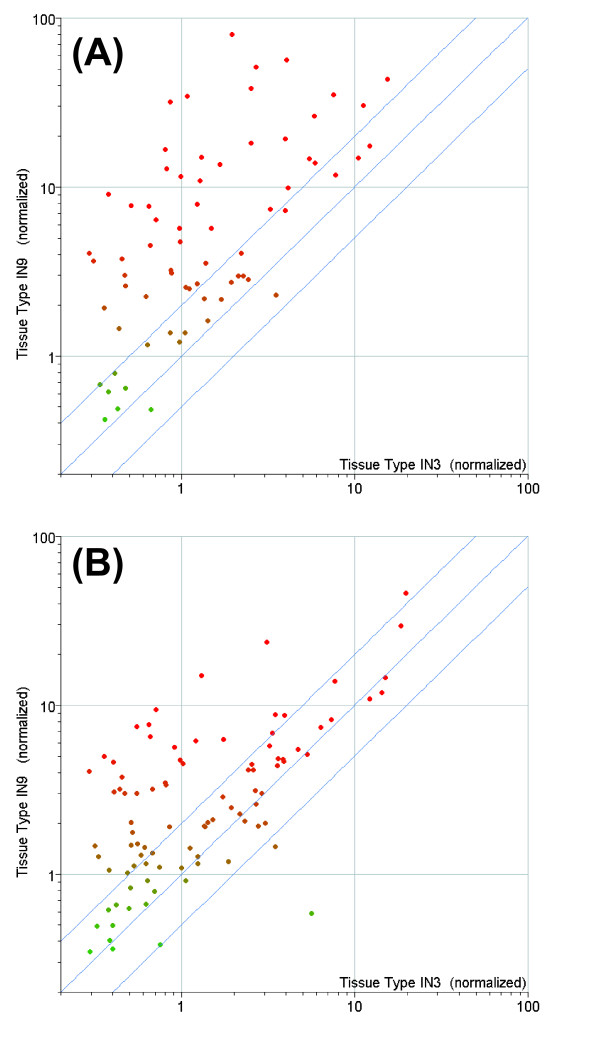
**Comparision of *Populus trichocarpa *genes on our array that is homologous to xylem-predominant genes from other studies**. Normalized intensity values for IN3 (internode 3) plotted against IN9 (internode 9). Upper and low diagonal lines show two-fold change ratio intervals. (A) Comparison to postulated Arabidopsis core-xylem genes [23]. (B) Comparison to xylem-predominant transcription factor genes [24].

We assessed our data for quantitative correspondence to published literature. Ko et al [23] have identified a core set of 52 Arabidopsis xylem-predominant genes through a global comparative transcriptome analysis of Arabidopsis "secondary growth" tissue, digital northern data from 45 organ/tissue types, and digital *in situ *data from vascular versus epidermal tissue. From these 52 "core" Arabidopsis genes we identified 69 homologous *P. trichocarpa *gene models on our array, and all but 8 genes in the core set had clear *P. trichocarpa *homologs (Additional file 1). More than 80% (51 genes) of expressed genes in this group (63) showed more than a 2-fold increase in the internode 9 sample (secondary growth dominant) when compared to the internode 3 sample (primary growth dominant: Figure 3A). A similar comparison of transcription factors associated with *Populus *and Arabidopsis secondary tissue [24] showed that only 2 genes out of the 82 expressed showed 2-fold or more down-regulation in internode 9 compared to internode 3 (Figure 3B); in contrast 41 genes (50%) showed more than a 2-fold increase. These results also support the biological accuracy and precision of data generated from our array.

### Global expression profile during stem development

Total RNA derived from the 5 stem internode development series described above was used for expression profiling with a Nimblegen microarray representing the whole annotated transcriptome at the time that the array was created. Within the 5 stem internode samples we could detect the expression of 43,283 genes out of the 65,915 represented on the array (9,995 unigenes derived from aspen EST sequences: [25]). To identify statistically significant, differentially expressed genes within the developmental series we used the EDGE software package [26,27]. A total of 4,279 *Populus trichocarpa *genes were identified as differentially expressed within the stem developmental series, when a Q value cutoff of 0.05 was employed; 87 genes were differentially expressed at a Q value cutoff of 0.01 (Additional file 2). To achieve a more stringent gene list we further filtered the 4,279 genes to remove weakly expressed genes (below 1.5 X the mean intensity of controls plus one standard error), and also removed genes with a fold-change below two. The resulting 3,016 genes were considered as differentially expressed in the manuscript, and used for further analysis and clustering. More than 15% of these genes were novel and had no significant BLAST returns ('No hit' cutoff e^-10^) in TAIR Arabidopsis or NCBI non-redundant sequence databases (June 2008). As the tissue sampling for this experiment was carried out at one time point during the active growth period, the set of differentially expressed genes might not reflect additional genes that have highly specific patterns of diurnal or seasonal regulation. To validate our differentially expressed gene list, we conducted qRT-PCR on three additional biological replicates using a subset of genes that were at the FDR cutoff of 0.05. Except for one out of the 9 genes tested, all showed a positive correlation between microarray and qRT-PCR expression patterns. The microarray and qRT-PCR derived fold-changes between elongation zone (IN2) and secondary growth dominant (IN9 or IN5) zones are illustrated in Additional File 3. The weakly regulated gene number one, identified as differentially expressed in the microarray dataset, did not show differential expression between IN2 and IN9 in the qRT-PCR analysis. As is commonly reported for microarray and qRT-PCR comparisons, the qRT-PCR expression fold-differences for all of the genes, except number 6 and 1, were considerably higher than that estimated from microarray data.

**Figure 4:**
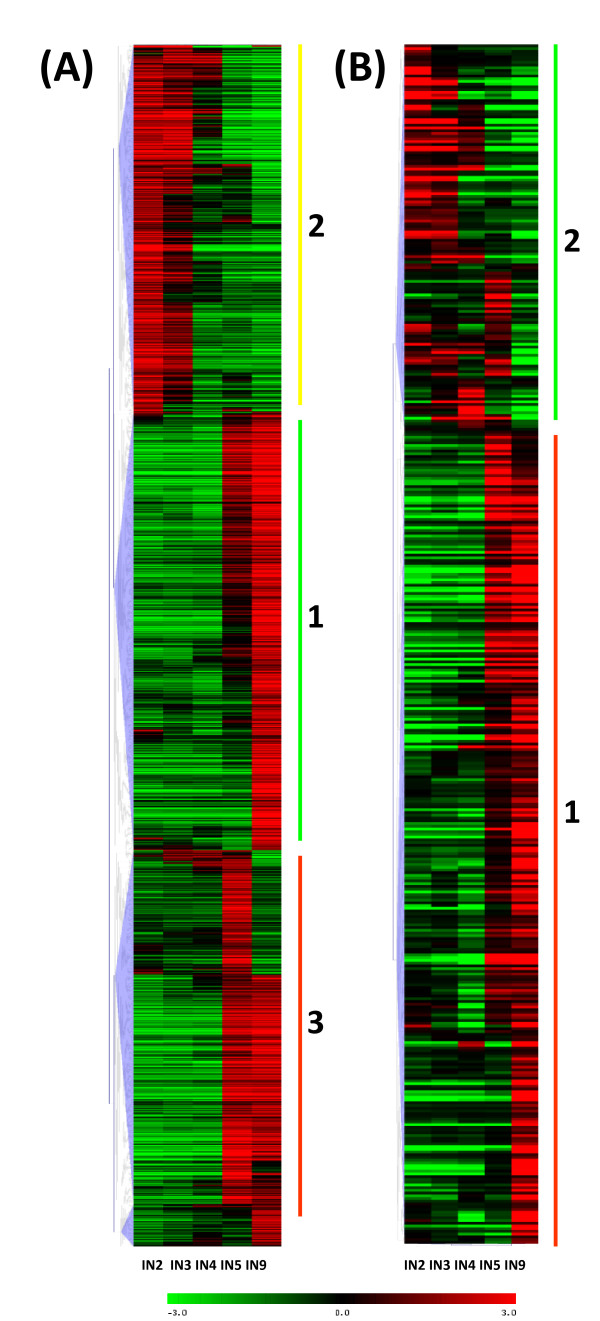
**Hierarchical clustering of all differentially expressed genes and transcription factor genes in stem internode tissue (IN2 - IN9)**. Mean centered expression levels are represented with green denoting down regulation, and red indicating upregulation. (A) All 3,016 differentially expressed genes in stem internode tissue (IN2 - IN9). The 3 major clusters of genes with similar expression patterns are marked with vertical lines and numbered 1 to 3. The expression values, gene models, and closest hit for each of these genes are listed in Additional file 2. (B) 439 transcription factor genes regulated during stem development. The two major clusters of genes with similar expression patterns are marked with vertical lines. Gene identities are illustrated in Additional file 5.

The clustering of all regulated genes (3016 genes) showed (Figure 4A) three main patterns of gene expression (gene order in additional file 2 correspond to the cluster diagram in Figure 4A). The collection of genes in cluster number 1 mostly showed increasing expression going from IN5 to IN9, with strongest expression in IN9. Cluster number 2 gene expression was dominant in the stem elongation and primary growth region (IN2, IN3), and tapered off as secondary growth began. Cluster number 3 genes showed strong upregulation in both IN5 and IN9, with its peak expression for most genes in IN5. This group also included some genes that showed more complex expression patterns, with upregulation in younger internodes (IN2, IN3) in addition to IN9.

Based on gene expression, the IN5 sample largely clustered with IN9 (Figure 4A), rather than with the IN4 sample that is adjacent in the stem. This correlated well with the switch to stem radial growth following activation of secondary growth in IN5. Thus, most genes are likely to be involved in processes specific to vascular cambium activity and secondary growth. Highly expressed genes in these 2 clusters include lignin biosynthetic genes, cell wall carbohydrate metabolic genes, arabinogalactan proteins, cytoskeleton-associated genes, and a number of transcription factor genes (discussed below). Other very active genes during secondary growth included transporter genes (peptide and amino acid transporters, aquaporins), proteases, lipid transfer protein and metallothionin-like genes.

The upper quarter of cluster 3 shows a sub-cluster of genes that are upregulated mainly in IN5. The latter group of genes could be associated predominantly with early stages of secondary growth activity of the cambium. As evident in IN5 stem cross section (Figure 1), the secondary wall development and lignification of xylem is just beginning in this sample, whereas well developed and lignified walls appear mainly in IN9 xylem. A highly expressed xyloglucan endotrans glycosylase belonging to this group has been reported to be highly expressed in aspen tension wood and is suggested to be involved in maintaining crosslinks between G and S layers of highly cellulosic tension wood fibers [28]. Several pectin methyl esterases and a UDP-glucose dehydrogenase that are likely involved in pectin and hemicellulose formation are also present within this group.

miRNAs have recently been recognized as critical post-transcriptional regulators of plant development and stress response. Our array contains only 47 miRNA precursors based on knowledge at the time of its creation; however, 234 miRNAs are now reported for poplar (miRBase miRNA database 2008 [29]). We were able to detect expression for 21 of these but only a few showed distinct regulation. miRNA164, 162a and 168 showed secondary development zone dominant expression. miRNA168 shows the strongest upregulation and is known to control Argonaute expression in Arabidopsis and poplar [30-32], which in turn is required for stem cell functioning, organ polarity, and root initiation in Arabidopsis [30,33].

Highly expressed genes within cluster number 2 include genes associated with primary cell wall synthesis, cell wall loosening/extension, and plant defense. Putative cell elongation-related genes within this group include expansins, extensins, a cell elongation protein similar to DWARF1, and an apyrase. DWARF1 [34] is a Ca/Calmodulin-regulated brassinosteroid biosynthetic gene required for cell elongation in Arabidopsis [35]. Apyrases are documented to be involved in cell growth regulation and accumulate in rapidly growing tissues with high auxin concentrations [36]. Thaumatin and osmotin-like proteins can have β-glucanase functions [37], and are also represented in the elongation zone dominant group. Biosynthetic genes that take part in plant secondary metabolite production are also well represented in this group and include several polyphenol oxidases, chlalcone synthases, and flavonol 3-hydroxylases. Phenolic and other defense-related genes have also been reported to be upregulated in young Sitka spruce stems [38]. In addition to the IN9-upregulated lipid transfer protein, several others are upregulated in the elongation zone. Lipid transfer proteins are abundant lipid binding-capable proteins with diverse proposed (but mostly unconfirmed) functions [39]. These include plant defense, signaling, cuticle biosynthesis, and cell wall loosening [40,41]. Most of the lipid transfer proteins upregulated in developing Sitka spruce stem were also found in the primary growth region [38].

### Gene function during early xylem maturation

To help assess the biological function of the regulated genes we identified during the early stages of cambial zone differentiation into xylem, we compared our results to previous studies of gene expression in aspen [42,43,9]. These studies used high resolution tangential cambial zone cryosectioning, but employed cDNA based microarrays that represented only ~30% of predicted gene models in poplar. Nonetheless, as discussed below, many genes were in common with the genes we identified.

We compared the genes showing the highest expression ratio in xylem maturation zone cryosections [42] to the differentially expressed gene set in our study. Of the 330 genes with the highest expression ratio (log2 ratio >2) in the initial maturation zone (MX1- [42]), we found 137 counterparts in our differentially expressed gene set (Figure 4A). Out of the 137 overlapping genes, 131 grouped in the IN5 and IN9 upregulated gene clusters (Figure 4A: Cluster 1 and 3), supporting their involvement in xylem maturation. The genes' closest hits and Arabidopsis homologs are listed in Additional file 4 and shows a dominant presence of genes associated with lignin biosynthesis, cell wall carbohydrate biosynthesis, and cell wall-associated proteins. Other major genes in common included transcription regulators (bZIP, MYB, ZF-C3HC4, LIM), hormone response genes (IAA, ethylene), and signaling genes (kinases, calmodulin binding), and cytoskeleton-associated genes (tubulins, microtubule-associated proteins).

### Functional roles of differentially expressed genes during stem development

**Figure 5:**
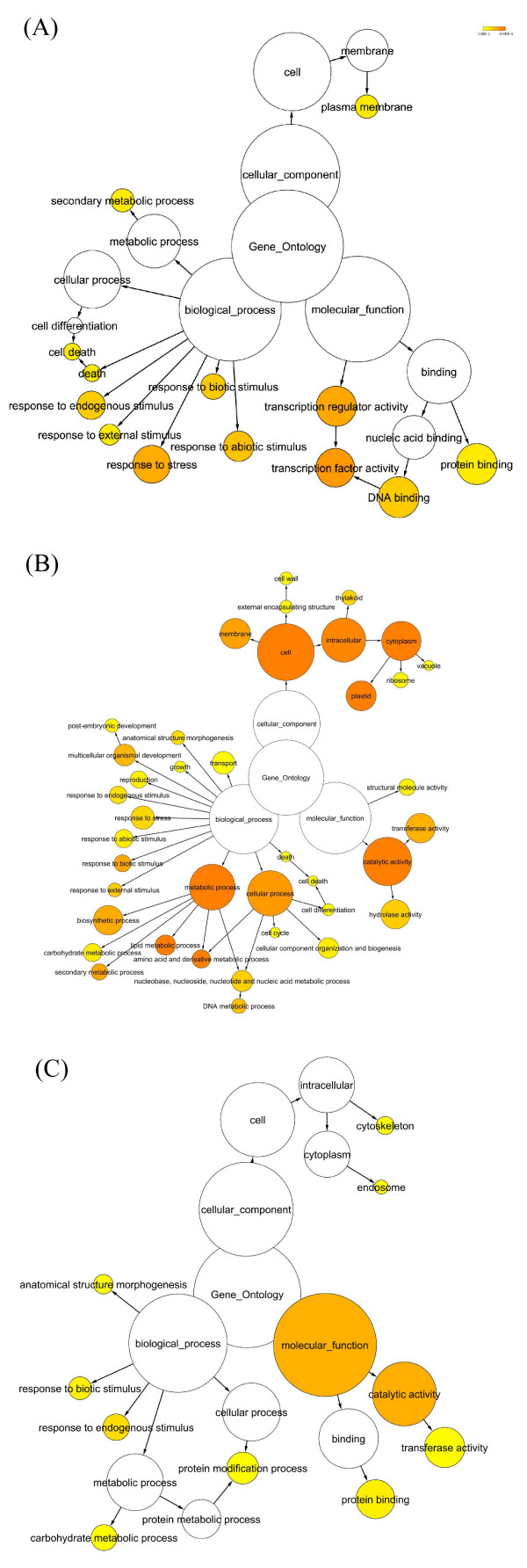
**Over-represented gene ontology categories during stem development**. Statistically over-represented GO categories based on each of the three main gene expression pattern clusters depicted in figure 4A. (A) Cluster 1 genes, (B) Cluster 2 genes, and (C) Cluster 3 genes. The circles are shaded based on significance level (yellow = FDR below 0.05), and the radius of each circle denotes the number of genes in each category.

To get a broader perspective on the function of differentially expressed genes, we analyzed for over-representation of gene ontology (GO) categories in each of the three main gene expression pattern clusters identified in figure 4A. The cluster 1 genes showed progressive upregulation with secondary growth (highest expression in IN9). Biological processes enriched in this group (Figure 5A) included several stress and stimulus response genes, secondary metabolic process genes dominated by lignin/phenylpropanoid biosynthetic genes, and cell death-associated genes. This enrichment should correlate with secondary wall development and terminal stages of xylem development that result in programmed cell death. The molecular function category is also highly enriched in transcription factor and DNA binding activity genes, as well as in protein binding activity genes related to cytoskeleton.

Cluster 3 genes also showed upregulation in both IN5 and IN9, but as opposed to cluster 1 peak expression was mainly in IN5. This contrast was visible in gene enrichment categories with primary cell wall and early stage secondary cell wall development-related categories showing dominance. The carbohydrate metabolic process and catalytic activity groups include cellulose biosynthesis, hemicelluloses/xylan biosynthetic genes as well as glycosyl group transferase activity and ubiquitin ligase activity-associated genes.

Cluster 2 genes, mainly upregulated in primary growth dominant IN2 and IN3 showed enrichment for many categories of biological processes, molecular functions and cellular components. Dominant biological process functional categories include active cell growth, development/morphogenesis/differentiation, and metabolic process-associated genes that reflect activities related to primary growth. Enrichment of nucleotide metabolism and DNA replication related categories could also reflect the high cell division activity during the initiation of cambium in the IN3 region. The secondary metabolism-enriched group includes flavonoid and anthocyanin biosynthetic genes.

### Transcription factor and regulatory genes

There are 2,576 transcription factors and regulatory genes in two poplar databases (http://dptf.cbi.pku.edu.cn/ and http://plntfdb.bio.uni-potsdam.de/v2.0/), and 2,542 of these were represented on the array. We found that 1801 genes were expressed at detectable levels in at least one of the 5 internode samples. Figure 4B shows the clustering of 439 transcription factors that are differentially regulated during stem development (see additional file 5 for gene identities). A recent related study on transcriptome changes during shoot organogenesis in *Populus *identified a comparable number (588) of regulated transcription factors [44]. We could recognize clusters upregulated in IN5-IN9, and in IN2-IN3. The majority of regulated transcription factors (69%) that were upregulated correlated with the onset of secondary growth.

**Figure 6:**
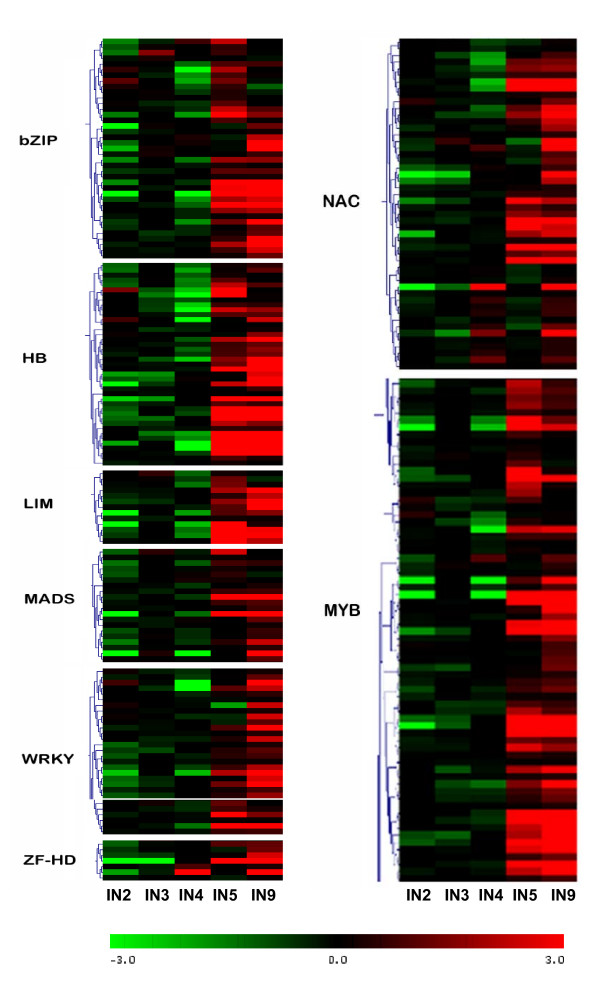
**Clustering of transcription factor classes implicated in secondary development (i.e., dominant expression in IN9 and IN5)**. Cluster diagram of all expressed family members and gene identities are in additional file 7.

Transcription factors that are predominantly expressed during vascular development and secondary growth are of considerable interest due to the economic importance of wood and wood fibers. As regulators that help to control response networks, they may be important tools for modifying wood or fiber qualities. Schrader et al. [9] have reported high resolution transcription maps of aspen wood forming tissue and have identified potential regulators of xylem differentiation. Many transcription factor and gene regulator classes have been reported in the literature to play a role in vascular and xylem development in different plant species (additional file 6). We selected all expressed genes belonging to these gene families in poplar and displayed their expression as a cluster diagrams (additional file 7). The sub-clusters of genes expressed in association with secondary growth of each gene family are illustrated separately in Figure 6.

With 297 identified members, MYB and MYB-like genes are the largest transcription factor family in poplar. Out of the 187 genes expressed in developing stems, more than 50% show secondary growth-associated expression patterns. MYBs are implicated as critical regulators of vascular differentiation and phenylpropanoid metabolism in plants, including the tree genera *Picea, Pinus, Eucalyptus *and *Populus *[45-48]. Overexpression of *Pinus *MYBs have resulted in ectopic lignification in tobacco [49]. The secondary wall biosynthesis-regulating Arabidopsis MYB46 has also been demonstrated to be a direct target of the secondary wall-associated NAC domain transcription factor SND1 [50], further elaborating the network of interactions. The *Populus trichocarpa *homologue of Arabidopsis MYB46, gw1.IX.3536.1 is also uprgulated in IN5, IN9 samples (Figure 6, Additional file 7). The complete group of secondary growth-associated MYB family members is identified in Figure 6.

Similar to MYBs, nearly 50% of expressed NACs, one of the largest plant-specific transcription factor families, were upregulated during secondary growth (Figure 6). Arabidopsis NACs, VND(Vascular-related NAC-domain), and NST(NAC secondary wall thickening promoting factor) groups of genes have preferred expression in vascular tissue, and have been identified as regulators of xylem vessel element differentiation and secondary wall formation in 'secondary growth' tissue of Arabidopsis [51,50,52]. The ectopic expression of *VND6, VND7, NST1, NST2 *and *NST3/SND1 *have induced transdifferentiation of various cells to produce thick secondary walls [51,53,51]. The 7 VNDs and 3 NSTs fall into separate phylogenetic branches but are grouped in the same subfamily of NAC proteins [51]. Although all putative *Populus *homologues of VND and NST are not regulated in our dataset, VND1 and VND2 homologues gw1.VII.3881.1 and gw1.57.51.1-JGI and NST3/SND1 homologue gw1.I.5485.1 are upregulated in IN5 and IN9 samples (Figure 6, Additional file 7). These genes were also specifically upregulated in xylem tissue in a *Populus *tissue-type dataset (results not shown), further supporting their role in vascular differentiation. Figure 6 illustrates more undescribed *Populus *NAC gene family members that show strong secondary growth-associated upregulation.

The homeodomain-containing superfamily of transcription factors participate in a wide variety of plant developmental processes. The leucine zipper-associated HD-Zip, wushel-related WOX, knotted-related KNOX, and zinc finger-associated ZF-HD homeodomain containing transcription factors have been associated with processes related to meristem function, organ polarity, and vascular development in several species [54]. Ko et al. [23] have shown that a hybrid aspen class III HD-Zip protein, Pt-HB1 was closely associated with wood formation and that it was tightly regulated over developmental and seasonal gradients, with highest expression during the active growing season. A *Populus trichocara *homologue of aspen Pt-HB1, estExt_Genewise1_v1.C_660759, was within the IN5, IN9 upregulated cluster (Figure 6, Additional file 7), and we also found additional homeodomain superfamily members (HB and ZF-HD in Figure 6) that were strongly upregulated during secondary growth.

bZIP transcription factors play critical roles in regulating plant defense responses and development [55]. In addition to being upregulated during *Eucalyptus *xylem development [56], the bZIP family has been identified as one of the transcription factor families most regulated during Arabidopsis stem development [57], with two specific candidates associated with xylem fiber differentiation. Poplar homologs of these two bZIP transcription factors are also represented within the many secondary growth-associated clusters of bZIP genes in Figure 6.

Although WRKY transcription factors are mainly implicated in regulating defense signaling [58], they have also been identified to be upregulated in Arabidopsis stem secondary growth and xylem tissue [59,60] and in aspen tension wood [61]. The WRKY family is highly diversified in plants. There were 21 poplar WRKY members that were upregulated during secondary growth, of a total of 104 total members in poplar. The functional study of this subset would help to expand knowledge of the diversity of WRKY developmental functions in plants.

There were 11 LIM genes associated with secondary development (Figure 6). LIM domain proteins in eukaryotes act as regulators of transcription, or in organization of the cytoskeleton. Tobacco NtWLIM1 binds F-actin and has been suggested to be involved in actin cytoskeleton stabilization [62]. In sunflower HaPLIM1 is found in actin-enriched cells, and exhibits non-specific DNA and RNA binding activity [63]. Tobacco NtLIM1 has been demonstrated to act as a transcription factor and is able to bind the conserved PAL-box found in promoters of many phenylpropanoid pathway genes [64]. Down regulation of NtLIM1 in tobacco also results in reduced lignin and decreased expression of phenylpropanoid genes. In poplar some LIM genes have been reported to be upregulated in tension wood [61]. Both the cytoskeletal component binding and transcription regulating activity of these genes can influence the lignin biosynthetic pathway, and thus influence secondary cell wall properties that affect wood quality and warrants further study into the secondary development associated LIM genes illustrated in Figure 6.

The few MADS box gene family members that were upregulated during secondary growth are illustrated in Figure 6. MADS box genes are well known for their involvement in reproductive development in many plants, and have also been shown to be important in vegetative development and dormancy in poplar [65,66]. The seasonal expression pattern of aspen MADS-box5 (*PTM5*) that is also represented in the secondary growth-associated cluster of poplar genes (Figure 6) is specific to developing vascular tissue, including vascular cambium. It interacts with other MADS box proteins and an actin depolymerizing factor, to perhaps play a major in control of woody tissue development [66].

An ethylene response element binding factor-like gene that belongs to AP2/ERF domain transcription factor class is one of the most strongly regulated genes in secondary growth dominant cluster 1 (Figure 4B). It has also been identified in aspen [20] as differentially expressed and phloem localized in secondary tissue. AP2/ERF family members are known to be involved in integration of jasmonic acid and ethylene signals in plant defense [67], but also have members (BOLITA, BABYBOOM) that affect cell expansion, proliferation and differentiation pathways in Arabidopsis [68-71].

### Carbohydrate active enzymes (CAZymes)

CAZymes http://www.cazy.org include the large families of glycosyl transferases (GTs) and glycosyl hydrolases (GHs), as well as two small families of polysaccharide lyases (PLs) and carbohydrate esterases (CEs). The full compliment of *Populus *CAZymes (1567) have been identified and reviewed in [72] and expression was analyzed for some families in [73]. Plants have more CAZymes than other organisms, and they are involved in diverse functions. These include assimilation of photosynthetic products, synthesis of various cell wall polymers, and synthesis and conversion of glycosylated compounds. Approximately 40% of the CAZymes identified in the *Populus trichocarpa *genome are represented in *Populus* EST collections [73]. Our data indicated that 71% of all CAZymes identified on the array are expressed during stem development (993 out of 1,392; Table 1). The expression of both GTs and GHs show relatively small clusters of genes that are associated with secondary growth (Additional file 8), and these could be gene family members associated with secondary wall metabolism. In contrast, a large proportion of GHs show elongation zone dominant expression patterns. Some members of this group could be involved in primary cell wall metabolism, cell wall loosening, and cell elongation. The poplar homologues of Arabidopsis FRA8, IRX8 and IRX9, and glucuronoxylan biosynthesis-secondary wall localized GT family genes [74,75] have also been localized to aspen secondary wall containing cells [76]. These three genes are grouped with the secondary growth-associated cluster of genes during stem development (Additional file 8).

**Table 1:** Carbohydrate catalyzing enzymes (CAZymes) expressed during stem development.

Major CAZyme Families	Total gene models (represented on poplar array)	Number of expressed transcripts
Glycoside hydrolases (GHs)	606 (567)	403
Glycosyl transferases (GTs)	825 (696)	497
Polysaccharide lyases (PLs)	39 (39)	26
Carbohydrate esterases (CEs)	98 (93)	67

Pectins include chemically diverse polymers that are major components of cell walls. They are important for wall plasticity and cell adhesion, and are major constituent of primary walls of poplar [77] that undergo substantial remodeling during development [78]. In addition to some GHs, members of CE and PE gene families are involved in pectin de-esterification/acetylation and pectin degradation. Siedlecka et al. [79] have shown that aspen pectin methyl transferases are involved in intrusive and symplastic cell growth in developing wood cells. The small clusters of PL and CE gene family members that are preferentially expressed in the elongation zone (Additional file 8) are possible candidates for control of pectin restructuring during stem elongation. The secondary growth-associated small clusters of PL and CE gene families are likely to be involved in pectin remodeling during the early stages of secondary wall biosynthesis and radial and intrusive growth of cambial derivatives. This would be applicable to the CE family members that show highest expression in IN5.

**Figure 7:**
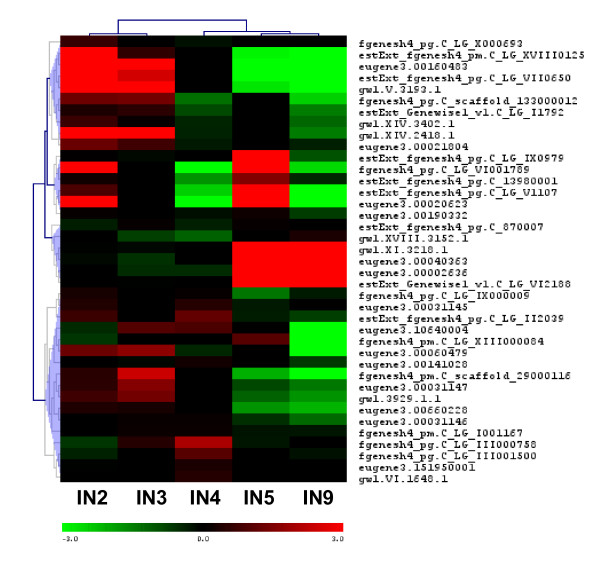
**Clustering and identities of CesA and Csl gene family based on gene expression**. Hierarchical clustering of expressed cellulose synthase (CesA) family and Ces like hemicelluloses-related genes (Csl) in stem internode tissue. Gene names/closest Arabidopsis hit information in Additional file 9.

We have illustrated the expression pattern of all expressed cellulose synthase (CesA) and hemicellulose-related cellulose synthase-like (Csl) genes in Figure 7. These were separated from other GT family members due to their importance in cellulose and hemicellulose biosynthesis. *Populus CesA *and *Csl *genes have been investigated earlier for family structure and expression in different tissue types [80-82], which led to the identification of a few members that are highly expressed in xylem tissue. Our results show a cluster of four genes that are highly expressed in the secondary growth zone that are homologous to Arabidopsis IRX1 (CesA8), IRX3 (CesA7) and IRX5 (CesA4) that are known essential components of cellulose synthesizing complex in Arabidopsis (Figure 7, Additional file 9). In addition, we could recognize a cluster predominantly expressed in the elongation zone and a cluster with dominant expression in IN5 in addition to elongation zone. These gene family members could preferentially be involved in primary cell wall synthesis during primary growth and secondary growth, respectively.

### Expansins and extensins

**Figure 8:**
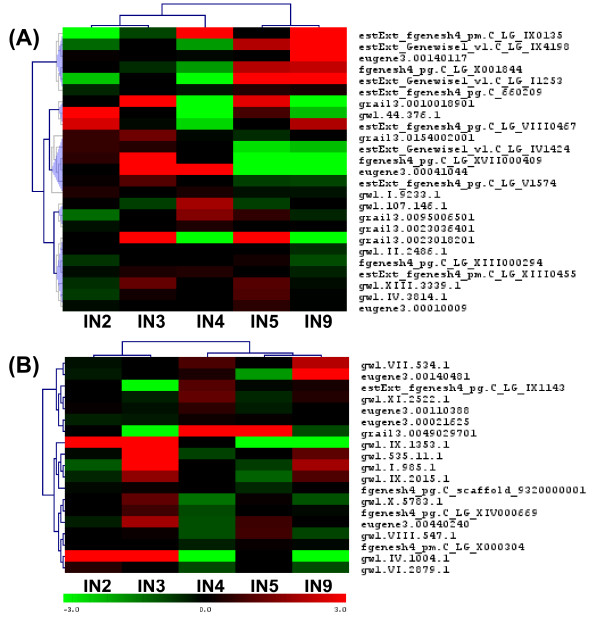
**Clustering and identities of expansin (A) and extensin (B) gene families based on gene expression**. Hierarchical clustering of expressed (A) expansin gene family (*EXPA, EXPB, EXLA, EXPLB*) and (B) extensin gene family. Gene names/closest Arabidopsis hit information in Additional file 9.

Expansins are considered one of the primary regulators of plant cell expansion and loosening [83,84]. α-expansins are abundant in aspen secondary xylem [85], and ectopic expression promotes cell expansion in both primary and secondary tissue of aspen [86]. Clustering showed a distinct group of five genes with secondary growth dominant expression, and a few other clusters with elongation zone only, or elongation zone and IN5 dominant expression (Figure 8A)

Extensins are cell wall structural proteins that take part in plant pathogen response and plant cell wall assembly, acting as scaffolds [87]. During poplar stem development a large proportion of extensin family members are preferentially expressed in the elongation zone, with IN3 sample showing the highest expression (Figure 8B).

### Cytoskeletal components

**Figure 9:**
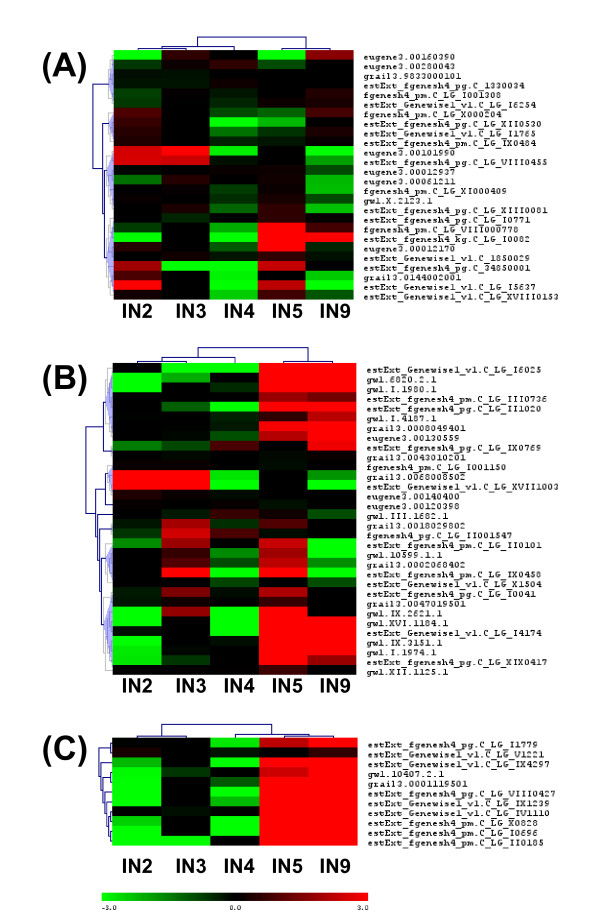
**Clustering and identities of cytoskeleton-related gene families based on gene expression**. Hierarchical clustering of expressed (A) actin (B) tubulin (C) actin depolymerizing factor (ADFs) gene families. Gene names/closest Arabidopsis hit information in Additional file 9.

The cytoskeleton plays an essential role in guiding and trafficking cellulose synthase complexes during cellulose deposition in secondary cell walls [88]. In developing xylem cells, microtubule bundles maintain the cellulose synthase complexes beneath the sites of secondary wall synthesis, while actin is required for delivery and rapid trafficking of complex-containing organelles around the cell [88]. Xylem fiber localized tubulin gene family members in *Eucalyptus *and *Populus *have also been reported to play a role in determining the cellulose microfibril orientation and angle, a determinant of wood fiber and timber quality [89,90]. Figure 9 illustrates the distinct groups of gene family members associated with secondary growth for actin, tubulin, and actin depolymerizing factor gene families. α- and β-tubulin genes that were upregulated in xylem and during tension wood formation [90] are within the secondary growth dominant group of genes in Figure 9. The similar upregulation of cell wall-associated cellulose synthase genes, and a Korrigan endoglucanase, in induced aspen tension wood [91] has led to suggest coordinated regulation of tubulin genes with cellulose biosynthesis machinery [90]. A smaller number of tubulin genes also showed preferential expression in the elongation zone.

### Fasciclin like-AGP

**Figure 10:**
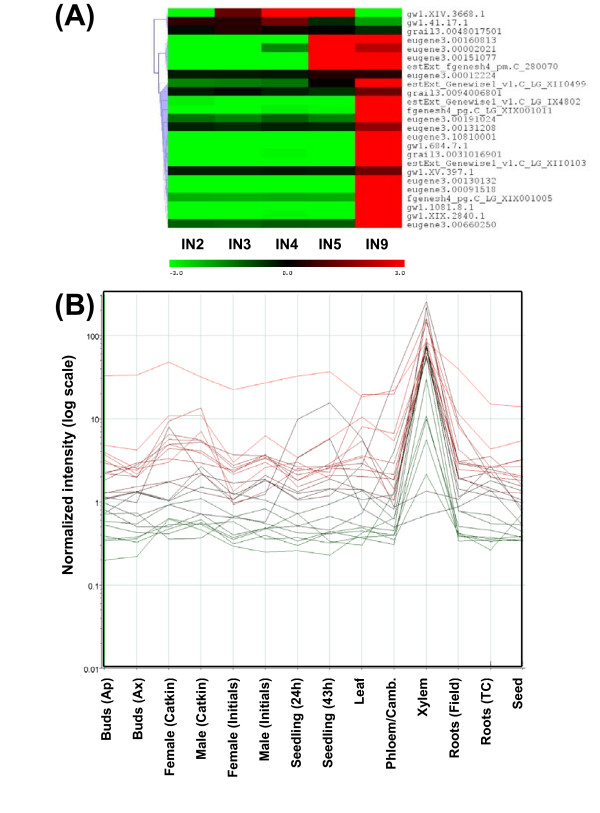
**Expression of fasciclin-like AGP gene family**. (A) Hierarchical clustering based on expression in stem internode tissue (B) Expression pattern in different *Populus trichocarpa *tissue types. Gene names/closest Arabidopsis hit information in Additional file 9.

Arabinogalactan proteins (AGPs) belong to the large family of hydroxyproline-rich glycoproteins (HRGPs) and also include extensins (discussed earlier), proline/hydroxyproline-rich glycoproteins, and lectins [92]. Fasciclin-like arabinogalactan proteins are a subclass of AGPs containing domains that resemble *Drosophila *fasciclin cell adhesion molecules [92]. Several Fasciclin like-AGPs have been associated with wood formation and are preferentially expressed in differentiating xylem in several species, including *Populus *[93,61,95]. Figure 10A illustrates that all gene family members except three are preferentially expressed in the wood forming IN9 sample. The lack of strong upregulation of this group of genes in the IN5 sample, and the exclusive expression of this group in xylem from a tissue type gene expression dataset (Figure 10B), illustrates its predominant role in xylem secondary wall biogenesis. Two genes showed high expression in the IN4 sample as opposed to IN9.

### Phenylpropanoid and lignin-related genes

**Figure 11:**
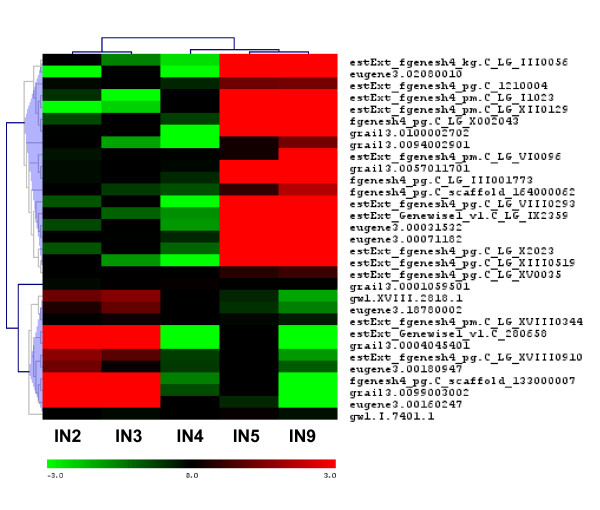
**Clustering and identities of phenylpropanoid genes based on gene expression**. Gene names/closest Arabidopsis hit information in Additional file 9.

An expression cluster diagram of phenylpropanoid genes identified in the poplar genome [2] is given in Figure 11. As expected, most lignin-related phenylpropanoid genes are predominantly expressed in the secondary growth zone. A small group that includes two phenylalanine amonnia lyases (*PAL3 *and *4*), a coumaroyl 3-hydroxylase CYP98A23 (*C3H *like), four hydroxycinnamoyl CoA shikimate/quinate hydroxycinnamoyltransferases (*HCT/HCQ 1, 2,4 *and *5*), and two 4-coumarate:CoA ligases (*4CL1*) are expressed predominantly in the elongation zone. These enzymes are involved in the early steps of phenylpropanoid metabolism and are likely involved in the biosynthesis of non-lignin components of phenylpropanoid derivatives such as phenolic esters and glycosides, condensed tannins, and flavonoid precursors. These are well known to have a variety of tissue abundances and complex responses to environment [96-98] that are mostly related to pathogen defense and protective functions.

## Conclusion

We have documented the transcriptome changes that occur during the transition from primary to secondary growth using sequential stem developmental stages. More than 3,000 genes were identified as differentially expressed, which is likely to be a conservative estimate due to the use of two biological replicates. Our results provide an extensive catalog of regulatory factors and cell wall biogenesis genes involved in stem elongation and secondary growth phase of tree development, whose potential applications include candidate genes for study of natural polymorphisms, targets for reverse genetic studies of tree development, and tools for marker-aided breeding or genetic engineering of tree growth and wood quality. The transcriptome patterns provided will also aid in making functional interpretations for the vast number of genes that have poorly known biochemical and developmental functions.

## Methods

### Plant material

Two healthy, rapidly growing, three-year-old *Populus trichocarpa *trees (clone Nisqually-1) at a field site in Corvallis, Oregon (USA) were selected for the study. The sample collection was performed on a clear day between 10 am and 11 am in August 2005. RNA from each of the two trees was used for separate array hybridizations, serving as separate biological replicates. Technical replications of hybridizations were not performed due to high platform technical consistency as determined in a previous study. Following measurement of internode lengths, the internodes (excluding the nodes) from the apical bud to the base of the shoot were excised into liquid nitrogen in the field and stored at -80°C. A 2 mm long segment of each internode was fixed in FAA for microscopy. Following dehydration these tissue segments were embedded in wax for sectioning (WAX-IT Histology Services, Vancouver). De-waxed stem sections were stained with Toluidine Blue-O [99] and photographed.

### RNA isolation

RNA was isolated using Qiagen RNeasy kit using the manufacturers protocols, incorporating the Qiagen "on column" DNAse I treatment to remove contaminating genomic DNA. The quality of total RNA was assessed by absorption at 260 nm/280 nm, gel electrophoresis, and via the Agilent Bioanalyzer (Agilent Technologies, USA).

### Microarray analysis

A poplar Nimblegen microarray http://www.nimblegen.com/ targeting 55,794 predicted genes from the *Populus trichocarpa *genome sequencing project, 126 mitochondrial and chloroplast gene models and 9995 unigenes derived from aspen EST sequences [25] were utilized for the experiment. Each gene model is represented by 3 replicated 60mer isothermal probes on the array. A total of 20 ug of quality tested total RNA from each sample was used for labeling. Array hybridization, quality control and data extraction were carried out by Nimblegen using their established microarray processing pipeline [100]. The complete microarray dataset has been deposited at NCBI GEO database http://ncbi.nlm.nih.gov/geo/ with Accession # GES13043

Microarray data were normalized across all the arrays used in the experiment using the Bioconductor - Robust Multiarray Averaging (RMA) protocol incorporated in the NimbleScan (Nimblegen Inc.) software. Duplicate readings were averaged following normalization. Background hybridization intensity for determining expressed genes was estimated using signal intensity of negative control probes on the array (*Escherichia coli *DNA sequence derived Ambion 'ArrayControl' spike probes, #1-AE000151, #4-AE000185, #6-M34825, #8-AE000184). The mean of normalized fluorescence intensity value plus one standard error of the negative controls of all arrays in the experiment was defined as the expression threshold. Gene Spring 7.3 (Agilent Technologies, USA), EDGE [26] and Microarray Software Suite-MeV4.0 http://www.tm4.org/ software were utilized for further data analysis and graphical display of results. Normalized data were analyzed using EDGE [26] to generate a list of differentially expressed genes with a false discovery rate cut off of 0.05. EDGE is a cross-platform, compatible open-source software running on the R statistical software package, and is based on a novel statistical methods that includes an Optimal Discovery Procedure and a time course methodology [101,26]. Clustering of gene expression was performed using hierarchical algorithm based on Pearson correlations and within-gene averaging. *Populus trichocarpa *genes homologous to Arabidopsis were identified using JGI annotation or Orthomap search tool http://orthomap.cgrb.oregonstate.edu. Functional enrichment/over-representation analysis was carried out using the network visualization program Cytoscape with GO plugins. For over representation determination, the Benjamini and Hochberg FDR-adjusted significance level cutoff was set at 0.05.

### Array quality and data precision

The A260/A280 ratios of RNA samples used for hybridizations ranged from 1.7 to 2.0. The absence of contaminating genomic DNA and integrity of RNA samples were examined by Agilent 2100 Bioanalyzer. RNA Integrity Numbers (RIN) of samples used for hybridization ranged from 8.1 to 10.0. The correlation coefficients (r^2^) of pair wise comparisons of normalized intensity values between the biological replicates ranged from 0.90 to 0.97. Data precision was estimated by calculating the mean coefficient of variation for the two biological replicates for the first internode sample (IN2) for all expressed genes (36,284). The mean standard deviation was 0.71 (14.8%), and the mean standard error was 0.50 (10.5%). To evaluate the correspondence between the biological replicates, in additional file 10 we present scatter plots of intensity values for all differentially expressed genes (FDR 0.05, 2 fold) between sample 1 (tree 1) and sample 2 (tree 2) at each internode. The graph shows that 99% of the genes fall within the 2-fold demarcation lines. The biological accuracy and precision of data generated from our arrays are further supported by the quantitative correspondence of secondary xylem marker gene expression from previously published literature described above (Figure 3).

### Quantitative RT-PCR

Real-time quantitative reverse transcription PCR (qRT-PCR) was employed to validate a select set of differentially expressed genes observed in our microarray study. Internode plant material was collected as described earlier from three individual trees different from what had been used for the microarray study. The sampling date was two days following the microarray study sampling date. Nine genes at the FDR cutoff (.049-.05) with low expression and fold-changes, and falling into the three main expression patterns (Figure 4A), were selected for verification. Elongation factor 1-beta (eugene3.00091463) was selected as the internal control gene [102] based on its consistency of expression level (mean SD = 11%) in the 5 stem tissue stages used in the study. RNA extraction, DNAse treatment, and quality control were performed as described above. cDNA synthesis from 1 ug of total RNA was carried out using a SuperScript III first-strand synthesis kit (Invitrogen) with oligo dT primers, as in the manufacturer's instructions. Primers were designed using Primer3 software (sequences in Additional file 11). Standard two-step RT-PCR and gel electrophoresis was used to evaluate primer pair efficacy and absence of genomic DNA contamination. Triplicate quantitative PCR assays per sample were performed using a Platinum SYBR Green Super mix (Invitrogen) with ROX reference dye according to the manufacturer's protocol. Dissociation curve analysis was performed at the end of each reaction to detect primer-dimers and multiple products. For relative quantification and comparison, we used the delta-delta-Ct method [103] with elongation factor 1-beta as the normalizing internal control gene. A 100% efficient amplification, resulting in a doubling of amplification product per cycle, was assumed (a requirement for the use of delta-delta-Ct method). This assumption was justified as only primer pairs showing an efficiency of 100% ± 5%, based on standard curves (pooled tissue type cDNA) were used for the experiments.

## Authors' contributions

PD conducted the experiment, analyzed the data, made biological and literature interpretations of the results, and wrote the first draft. SHS conceived of, directed, and co-funded the project; helped to direct data analysis and interpretation; and edited the manuscript. AB wrote the grant that funded the array hybridizations, provided the tissue type dataset for analysis, and helped to edit the MS. All authors read and approved the final manuscript.

## Supplementary Material

Additional file 1 **Core-xylem genes**. *Populus trichocarpa *genes on the array that are homologous to core xylem-predominant genes in Arabidopsis [23]. Click here for file

Additional file 2 **Differentially expressed genes**. *Populus trichocarpa *genes that were differentially expressed (3016 genes). Gene order as in the Figure 4(A) cluster diagram. Click here for file

Additional file 3 **qRT-PCR validation of microarray results**. qRT-PCR expression comparison of 9 selected genes from the microarray data; (1) estExt_Genewise1_v1.C_1240151,(2) estExt_Genewise1_v1.C_1700234,(3) gw1.XVI.2910.1,(4) eugene3.00121203,(5) gw1.236.44.1,(6) gw1.IX.897.1,(7) fgenesh4_pg.C_LG_III000388,(8) fgenesh1_pg.C_scaffold_385000002 and (9) estExt_fgenesh4_pm.C_LG_III0736. Genes 1 to 3 are from the gene cluster with high IN9 expression (Figure 4); genes 4 to 6 are from the IN2 high expression cluster, and genes 7 to 9 are from the IN5 high expression cluster. The fold-changes for genes 1 to 3 are between those found for IN9 and IN2, for genes 4 to 6 are between those found for IN2 and IN9 and for genes 7 to 9 are between those found for IN5 and IN2. The error bars on the qRT-PCR data column represent the standard deviation between the averages of the three biological replicates. Click here for file

Additional file 4 **Xylem maturation genes**. IN5 and IN9 upregulated genes that overlap with initial xylem maturation zone (cryosections MX1: [42]) over expressed genes (log2 expression ratio >2). Click here for file

Additional file 5 **Differentially expressed *Populus trichocarpa *transcription regulators**. Gene order as in the Figure 4(B) cluster diagram. Click here for file

Additional file 6 **Transcription regulators and vascular development**. Literature on transcription factor and regulator classes implicated in vascular and xylem development. Click here for file

Additional file 7 **Expression pattern of transcription factor families**. Cluster diagram of regulated transcription factor families implicated in vascular and xylem development. Click here for file

Additional file 8 **Expression pattern of carbohydrate catalyzing enzymes (CAZyme)**. Cluster diagrams of expressed CAZyme gene family members: Glycosyl transferases (GTs), glycosyl hydrolases (GHs), polysaccharide lyases (PLs), and carbohydrate esterases (CEs). Click here for file

Additional file 9 **Gene identities for Figures **7, 8, 9, 10 and 11. Gene lists, and gene names and/or closest hit information corresponding to Figures 7, 8, 9, 10 and 11. Gene order is as in cluster diagrams. Click here for file

Additional file 10 **Microarray replicate correspondences**. Scatter plots of intensity values for all differentially expressed genes (FDR < 0.05, 2-fold) between biological replicate sample 1 and replicate sample 2 at each internode. Click here for file

Additional file 11 **Primers used for qRT-PCR verification**. The sequence, length, tm, GC%, and product sizes of primers used for qRT-PCR. Click here for file
