# Supplementary figures and images for: Genome-wide transcriptome analysis of the transition from primary to secondary stem development in Populus trichocarpa
Source: BMC Genomics. 2010 Mar 4;11:150. doi: 10.1186/1471-2164-11-150 (PMC2846914; doi:10.1186/1471-2164-11-150)

## Slide 1
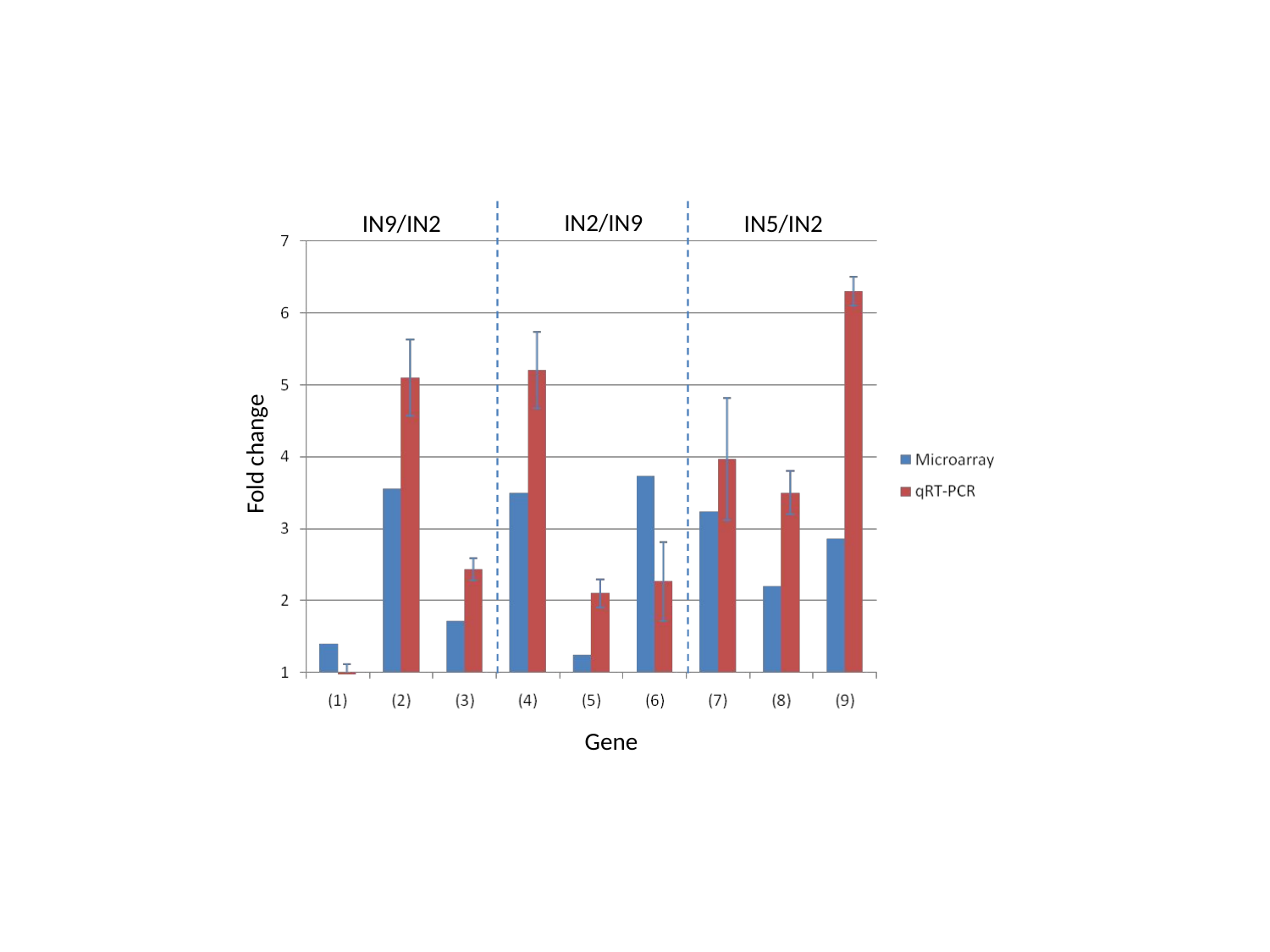

IN2/IN9
IN9/IN2
IN5/IN2
Fold change
Gene

Supplement: Additional file 3 — qRT-PCR validation of microarray results. qRT-PCR expression comparison of 9 selected genes from the microarray data; (1) estExt_Genewise1_v1.C_1240151,(2) estExt_Genewise1_v1.C_1700234,(3) gw1.XVI.2910.1,(4) eugene3.00121203,(5) gw1.236.44.1,(6) gw1.IX.897.1,(7) fgenesh4_pg.C_LG_III000388,(8) fgenesh1_pg.C_scaffold_385000002 and (9) estExt_fgenesh4_pm.C_LG_III0736. Genes 1 to 3 are from the gene cluster with high IN9 expression (Figure 4); genes 4 to 6 are from the IN2 high expression cluster, and genes 7 to 9 are from the IN5 high expression cluster. The fold-changes for genes 1 to 3 are between those found for IN9 and IN2, for genes 4 to 6 are between those found for IN2 and IN9 and for genes 7 to 9 are between those found for IN5 and IN2. The error bars on the qRT-PCR data column represent the standard deviation between the averages of the three biological replicates. [file 1471-2164-11-150-S3.PPT]

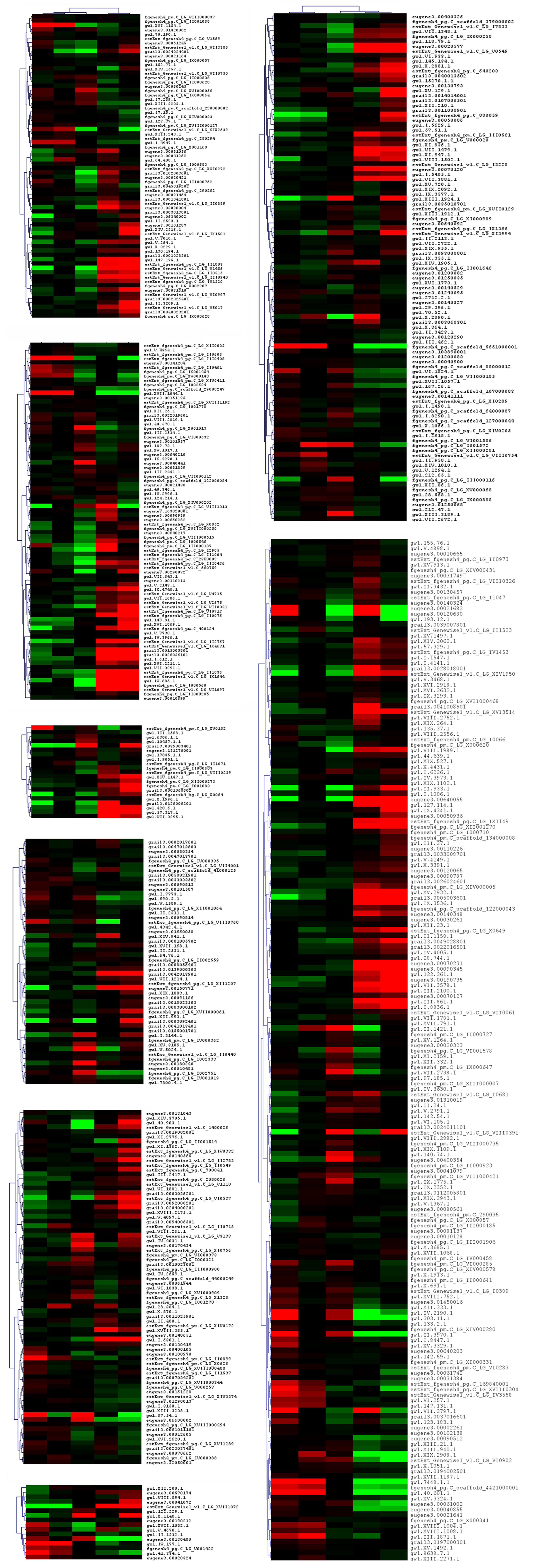

Supplement: Additional file 7 — Expression pattern of transcription factor families. Cluster diagram of regulated transcription factor families implicated in vascular and xylem development. [file 1471-2164-11-150-S7.JPEG]

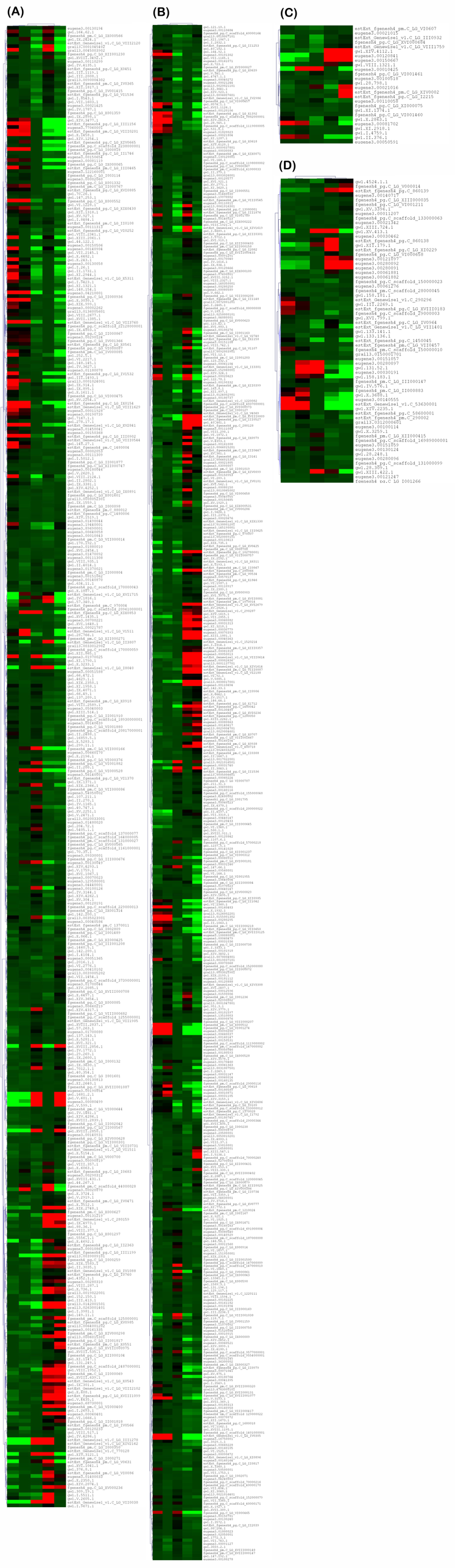

Supplement: Additional file 8 — Expression pattern of carbohydrate catalyzing enzymes (CAZyme). Cluster diagrams of expressed CAZyme gene family members: Glycosyl transferases (GTs), glycosyl hydrolases (GHs), polysaccharide lyases (PLs), and carbohydrate esterases (CEs). [file 1471-2164-11-150-S8.JPEG]

## Slide 1
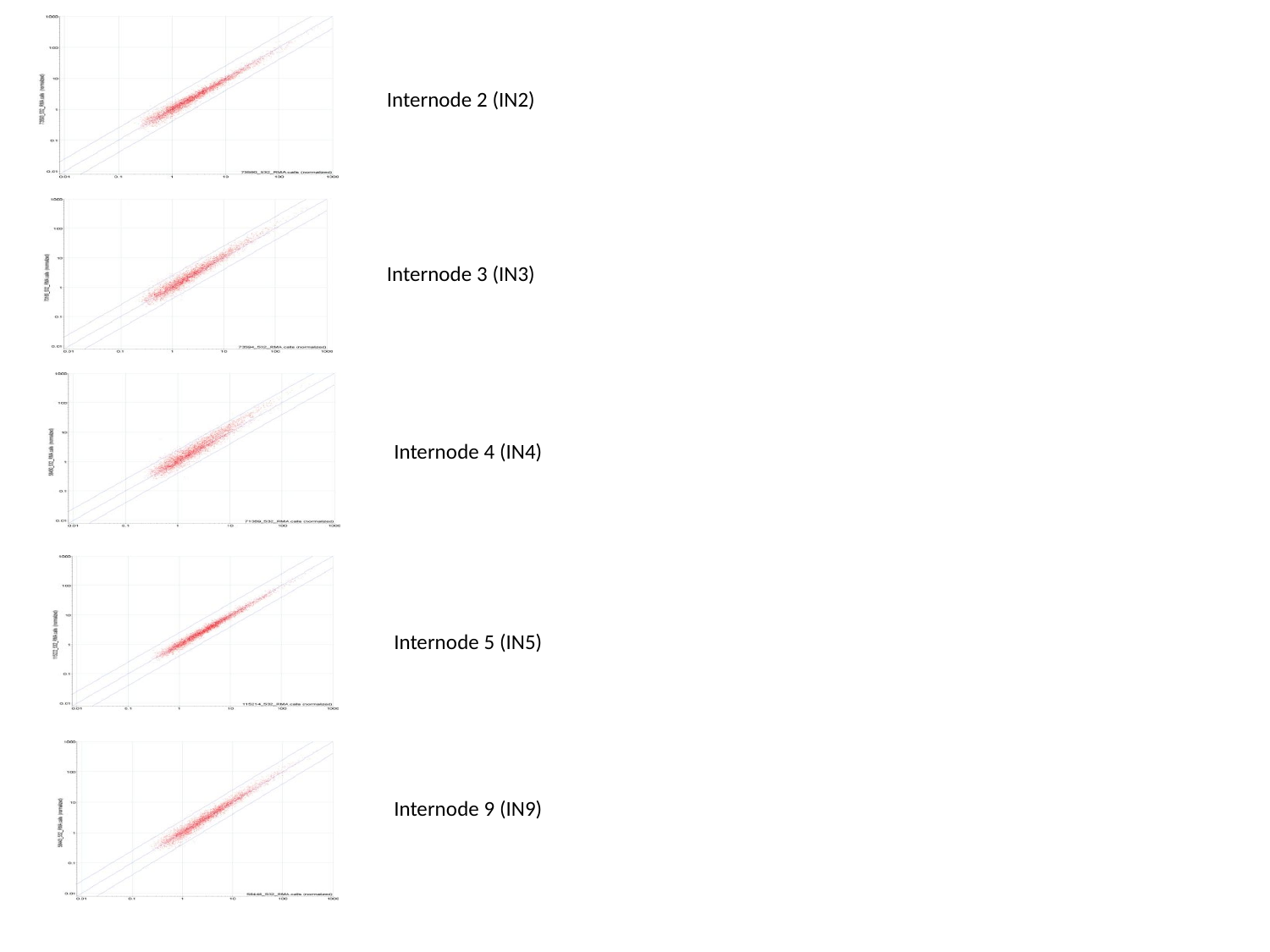

Internode 2 (IN2)
Internode 3 (IN3)
Internode 4 (IN4)
Internode 5 (IN5)
Internode 9 (IN9)

Supplement: Additional file 10 — Microarray replicate correspondences. Scatter plots of intensity values for all differentially expressed genes (FDR < 0.05, 2-fold) between biological replicate sample 1 and replicate sample 2 at each internode. [file 1471-2164-11-150-S10.PPT]
