# Supplementary material for: Genome-wide transcriptome analysis of the transition from primary to secondary stem development in Populus trichocarpa
Source: BMC Genomics. 2010 Mar 4;11:150. doi: 10.1186/1471-2164-11-150 (PMC2846914; doi:10.1186/1471-2164-11-150)
Supplement: Additional file 6 — Transcription regulators and vascular development. Literature on transcription factor and regulator classes implicated in vascular and xylem development. [file 1471-2164-11-150-S6.DOC]

Table 1

| **Species** | **Class** | **Reference** |
| --- | --- | --- |
| *Arabidopsis* | AP2/EREBP, MYB,bZIP,HD,C3H | Elthing et al 2005. |
|  | HD-ZIPIII, NAC,MYB,bHLH,WRKY | Zhao et al. 2005 |
|  | MYB,AP2/ERF,WRKY,NAC,HD,AUX/IAA,ARF | Ko et al. 2004 |
|  | ZF,HD,AP2,MYC,NAC,MYB | Ko and Han 2004 |
|  | NAC,MYB,bHLH,HD,ZF | Kubo et al. 2005 |
|  | NAC | Zhao et al. 2008 |
| *Populus* | HD-ZIPIII, MYB | Hertzberg 2001, Ko et al. 2006 |
|  | ZF,WRKY,LIM | Andersson-Gunneras et al. 2006 |
|  | MADS, LIM | Cseke et al. 2007 |
| *Pinus* | MYB | Yang et al. 2003 |
|  | Hap5a |  |
| *Picea* | MYB,WRKY, ARF-4,bHLH,HD-ZIPIII | Friedmann et al. 2007 |
| *Eucalyptus* | HD, bZIP, ZF | Paux et al. 2004 |
| *Zinnia* | ARF,HD-ZIPIII,NAC.AUX/IAA | Milioni et al. 2002 |
|  | ARF,HD-ZIPIII, NAC | Demura et al 2002 |
| *Hordeum* | HRT | Ivanov et al. 2008 |
